# The role of anthropogenic disturbance and invasion of yellow crazy ant in a recent decline of land crab population

**DOI:** 10.1038/s41598-021-91732-z

**Published:** 2021-06-10

**Authors:** Ching-Chen Lee, Ming-Chung Chiu, Chun-Han Shih, Chin-Cheng Scotty Yang, Hung-Chang Liu, Chung-Chi Lin

**Affiliations:** 1 Department of Biology, National Changhua University of Education, No. 1, Jin-De Rd., Changhua City, 50007 Taiwan; 2 Department of Entomology, Virginia Polytechnic Institute and State University, Blacksburg, VA 24061 USA; 3 Department of Entomology, National Chung Hsing University, Taichung, 402204 Taiwan; 4 Unaffiliated, 53, Chenggong 11th St., Jhubei City, Hsinchu County, 302 Taiwan

**Keywords:** Invasive species, Entomology

## Abstract

The yellow crazy ant, *Anoplolepis gracilipes* was first reported in Kenting National Park, Taiwan, in the 2000s, raising the concern of this invasive ant’s potential effects on the native land crab populations. We investigated the distribution and abundance of *A*. *gracilipes* and its adverse effect on the land crab populations at four land crab hotspots within the park. Our survey results indicated that *A*. *gracilipes* was widespread and abundant at three of the study sites (Hsiangchiaowan, Shadao, and Natural Spring), while the distribution was much more confined at the fourth site (Houwan). Land crab populations experienced a notable decline at all the study sites except Houwan, indicating an inverse relationship between the population of land crabs and distribution/abundance of *A*. *gracilipes*. Combining the results of visual observations, the decline of land crab populations can be attributed, at least partially, to direct attacks by *A*. *gracilipes* on land crabs in their natural habitat and during the breeding migration season. Generalized additive model showed that worker abundance of this ant is associated with human disturbance levels, suggesting that anthropogenic disturbance may have contributed to the decline in land crab populations via promoting the abundance of *A*. *gracilipes*.

## Introduction

Social insects, particularly ants, are one of the most successful invasive groups due to their highly adaptable nature coupled with unique characteristics that significantly contribute to their ecological dominance (e.g., high reproductive rate, polygyny, and colony budding)^1,2^. In general, invasive ants are highly aggressive and often outcompete native species in their introduced areas^3–5^, negatively affecting the diversity and abundance of native invertebrates^1,6,7^, vertebrates^8–10^, and ecological services^11^.

Invasive ants can generally thrive in anthropogenically disturbed environments or human-modified areas such as landscape areas, forest edges, and agricultural fields^12^. The successful colonization of invasive ants could be facilitated by human activities for several reasons including: (1) anthropogenic habitat modification creates a conducive physical environments that favour the establishment of invasive ants (e.g., accessibility to food source, elevated soil moisture, relatively stable microclimatic conditions)^13–15^; (2) habitat modification or conversion negatively affects native ant assemblages that confer biotic resistance to species invasion^16,17^; (3) propagules of invasive ant species are typically first introduced in areas with considerable amounts of human activity, especially for those species that reproduce primarily by budding and depend mainly on human-mediated jump dispersal to spread to geographically distant areas^1,18^.

The yellow crazy ant, *Anoplolepis gracilipes* most likely originates from Southeast Asia^19–21^ and has spread to Australia, the Caribbean, and the islands of the Indian and Pacific Oceans via human commerce^21^. The yellow crazy ant forms polygynous supercolonies in which individuals exhibit limited aggression towards conspecific individuals from physically separated nests^22^. The yellow crazy ant is an omnivorous ant species that preys on a wide range of insects, crustaceans, arachnids, myriapods, and molluscs^23^. Owing to its numerical abundance, *A*. *gracilipes* is known to impact the taxonomic composition and abundance of invertebrate communities in its invaded regions^24–27^. The most striking example is the invasion of *A. gracilipes* on Christmas Island that leads to a major decline in the population of an endemic red crab (*Gecarcoidea natalis*), an increased rate of seedling recruitment and litter accumulation, and alteration of the island’s forest landscape structure^11^. *Anoplolepis gracilipes* can also harm vertebrates; it is known to kill newly born domestic animals and cause nesting failure in some bird species^8,23^.

**Figure 1:**
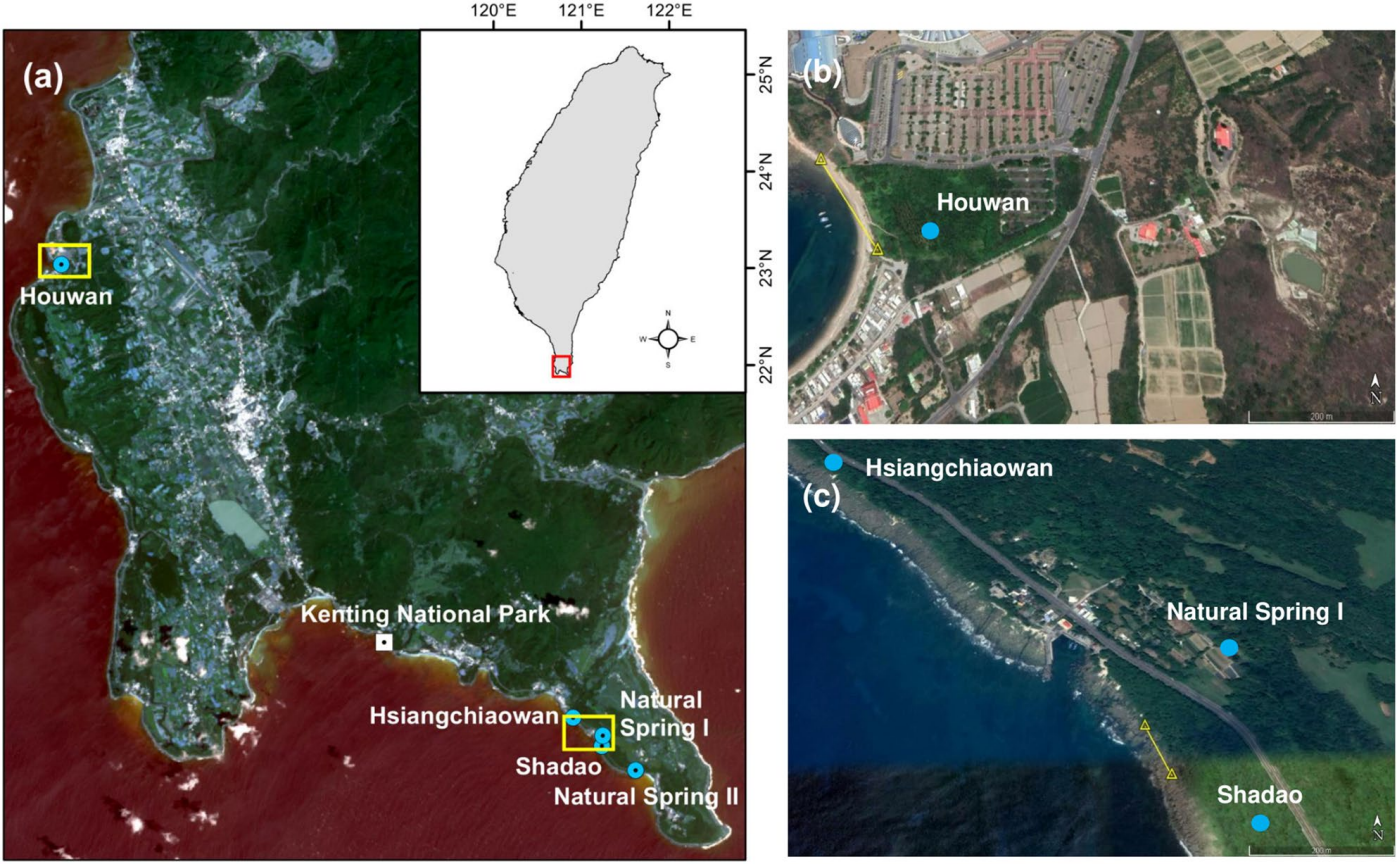
(**a**) Satellite images of Kenting National Park and locations of the study sites indicated as filled blue circles. Monitoring of ovigerous females was carried out near the intertidal zone (marked with yellow line) in (**b**) Houwan and (**c**) the Hsiangchiaowan–Shadao area. For (**a**), map was created using ArcGIS v.10.2, URL: http://www.esri.com/software/arcgis/arcgis-for-desktop. The background image (13 September, 2015) of panel (**a**) was derived from Landsat 8 Operational Land Imager using GloVis platform (https://glovis.usgs.gov/).; For (**b**), the satellite imagery is from Google Earth Pro (Map data 2019 Google; https://www.google.com/maps/@22.0429108,120.703344,847m/data=!3m1!1e3); For (**c**), the satellite imagery is from Google Earth Pro (Map data 2019 Google; https://www.google.com/maps/@21.9251393,120.8372065,1213m/data=!3m1!1e3).

Although the earliest record of *A*. *gracilipes* in Taiwan can be dated back to as early as 1909^28^, data regarding long-term population monitoring of this invasive ant in Taiwan has been scarce until the early 2000s. For example, this ant was first reported in Kenting National Park (Pingtung County, Taiwan, Fig. 1a), one of the largest national parks in Taiwan, in 2000, and multiple follow-up surveys in 2012 and 2013 have shown that this ant remained at a low population density in the national park^29,30^. Kenting National Park is well-known for housing an extremely high diversity of land crabs, with a total of 86 land crab species belonging to nine families recorded to date^31–37^. However, a 2015 survey revealed a major decline in land crab populations at virtually all land crab hotspots in the park (e.g., Hsiangchiaowan–Shadao, Fig. 1c), with one exception involving a hotspot site named “Houwan” (Fig. 1b) where the land crab populations continued to grow since a land crab survey initiated in 2003^38^. Considering that abiotic conditions (e.g., landscape and weather) have remained largely similar over years, biotic factors such as the yellow crazy ant may have contributed to the observed decline of land crab populations in these hotspots. Several field observations seem to support our speculation. For example, no cases of attacks on the land crabs by *A*. *gracilipes* were reported during an intensive land crab survey from 2012 to 2014. Since 2015, Liu^38^ observed numerous occasions of such attacks across different land crab hotspot sites in the park, suggesting that there must have been frequent encounters between the land crabs and *A. gracilipes* as a result of expansion of the ant’s territory in most of the land crab hotspots (except Houwan).

We therefore hypothesized that land crab populations would be high where populations of *A*. *gracilipes* were low, and exhibited decline where ant populations were high. To test this hypothesis, we conducted a series of field surveys to understand correlation of land crab population dynamics (i.e., from 2015 to 2017) and status of *A*. *gracilipes* (i.e., distribution and abundance) within Kenting National Park. Additional statistical modelling was performed to investigate whether abundance or distribution of this invasive ant was associated with the extent of human disturbance. These data allow us to further assess the link between intensity of human interference and establishment and/or spread of *A*. *gracilipes*.

## Methods

### Study area profile

Kenting National Park (21° 90′ N, 120° 80′ E) is located at the southernmost tip of Taiwan, covering approximately 17,731 ha of land and 14,900 ha of adjacent ocean. The park is surrounded by the sea and possesses marine, coral reef, and coastal forest ecosystems that provide an excellent habitat for land crabs. The climate of this region is tropical with humid summers and dry winters. Precipitation is usually concentrated from May through September.

### Study site and sampling points

A total of four land crab hotspots in Kenting National Park were selected, namely Houwan, Hsiangchiaowan, Shadao, and Natural Spring (consisting of Natural Spring I and II subareas) (Fig. 1a), each with 60 sampling points to assess the distribution and abundance of *A. gracilipes*. In Natural Spring, however, 60 sampling points were respectively set up in each subarea, resulting into a total of 120 sampling points in the site. Each sampling point was spaced at least 10 m apart from each other within the same study site. The sampling points in Houwan were distributed around coastal forest and one internal walking trail, whereas the sampling points in both Hsiangchiaowan and Shadao were distributed around internal walking trails (surrounded by fossilized coral reefs) and 1-km and 1.6-km roads, respectively. The sampling points in Natural Spring were distributed around an approximately 1.6-km road and agricultural land (e.g., predominantly comprised of crop vegetation, pond and farmhouse).

### Distribution and abundance of *A. gracilipes*

**Figure 2:**
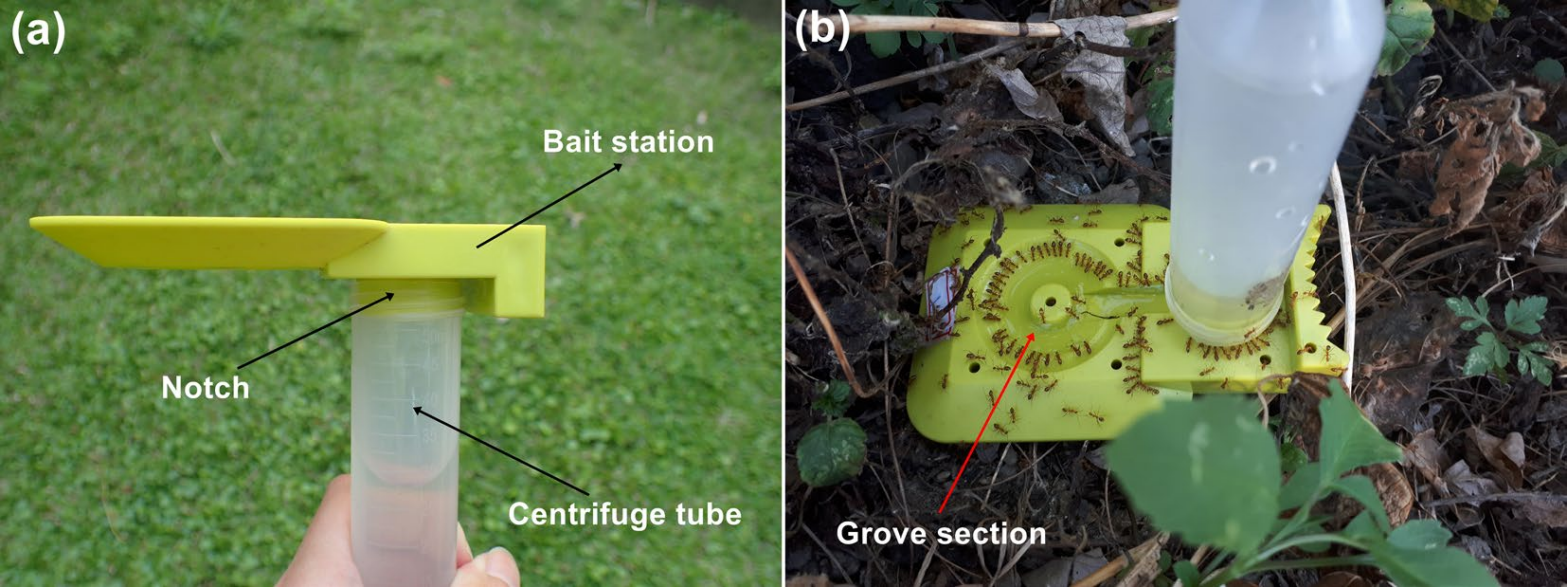
(**a**) Liquid bait station and (**b**) *Anoplolepis gracilipes* workers feeding on 10% sucrose solution.

Fieldwork was conducted once every 2 months from February to December 2017 (i.e., February, April, June, August, October, and December). To determine the distribution and abundance of *A*. *gracilipes*, we deployed liquid bait stations^39^ at each sampling point in all study sites (Fig. 2). As shown in Fig. 2a, a liquid bait station comprised a centrifuge tube filled with 30 mL of 10% sucrose solution (w/w), which was attached inverted to the notch of an upside-down station. The tube was inverted to allow the bait solution to fill up the grove section where numerous ants were able to feed on the solution simultaneously (Fig. 2b). We used ant activity counts (i.e., number of foraging ants on the bait station 30 min after deployment) to quantify worker abundance of *A*. *gracilipes*. Digital images of the foraging ants on the bait stations were captured using a camera (Tough TG-3, Olympus, Japan). The survey was conducted from 5:00 to 10:00 AM and from 2:00 to 6:00 PM at all study sites. Ant activity was not assessed from 11:00 AM to 2:00 PM as the ground temperature typically exceeded 35 °C during this period (especially during summer) and prevented most ants from foraging^40^. Worker abundance at each bait station was counted based on the digital images and was scored according to the following categories: 0 = no ants; 1 = 1–5 ants; 2 = 6–20 ants; 3 = 21–50 ants; 4 = 51–100 ants; 5 =  > 100 ants. The abundance counts were pooled and averaged by sampling date (i.e., month) and study site. Bait stations with no *A*. *gracilipes* captured were not included in the subsequent statistical analyses (see “Statistical analysis” section).

### Diversity and abundance of land crabs

Note that different crab species were focused during our field observations as to reflect the fact that dominant land crab species vary across our study sites (see below for more details).

#### Houwan

To allow for a fair comparison between the current study and Liu^38^, we followed the same survey technique, timing, location, and protocol used by Liu to understand the fluctuation of land crab populations over time (i.e., 2015–2017). The survey was conducted in August 2017 near the Houwan area of Kenting National Park. We patrolled near the intertidal zone (marked with yellow line in Fig. 1b, approximately 200 m long) after sunset every day from the first day until the sixth day of the lunar month as mass migration of land crabs usually occurs in conjunction with spring tides (i.e., during the full moon and new moon) that can quickly flush larvae into the ocean to avoid predation by other animals^41^. The species identity and number of ovigerous females migrating to the coastline to release larvae were recorded. We focused on *C. carnifex*, *C*. *hirtipes*, *S. intermedium*, and *S. impressus* because they are among the most dominant species in this area^38^. Ovigerous females were marked with marking pens on the carapace after being recorded to prevent repeated sampling. As the data on *A*. *gracilipes* from the year 2015 in all land crab hotspots are limited, additional observations of ant attack during land crab survey can serve as supplementary evidence that allows empirical characterization of the ant’s effects on the land crab population possible. Hence, during the survey, we also recorded the number of ovigerous females under attack by *A*. *gracilipes*, if any.

#### Hsiangchiaowan–Shadao

The survey of Hsiangchiaowan–Shadao was conducted in September 2017. We patrolled near the intertidal zone (marked with yellow line in Fig. 1c, approximately 100 m long) from the twenty-sixth day until the twenty-ninth day of the lunar month (from 2:30 to 5:00 AM). We focused on the abundance of a single dominant land crab species, *M. aubryi*^42^. Similar to the Houwan survey, the number of ovigerous females encountered and the number of ovigerous females under *A*. *gracilipes* attack were recorded.

### Spatial distribution of *A. gracilipes* in relation to anthropogenic disturbances

In order to examine the influence of anthropogenic disturbances on the distribution or abundance of *A*. *gracilipes*, the proportion of human-disturbed land cover was utilized as an indicator of intensity of anthropogenic disturbance. First, a circular buffer of 100 m radius of each sampling site was generated using Buffer function. Second, the circular buffer of each sampling point was intersected with land-use data obtained from the National Land Surveying and Mapping Center in Taiwan (https://maps.nlsc.gov.tw/)^43^. The analysis was conducted based on Buffer function and Intersect tool respectively in ArcGIS v.10.2^44^. Then the area and proportion of anthropogenic and human-disturbed land cover, such as agriculture, building, public facilities, recreation, transportation, etc., could be calculated.

### Statistical analysis

Student’s *t*-test was performed to compare the mean number of ovigerous females recorded per day for each dominant land crab species between year 2015 and 2017, at Houwan and Hsiangchiaowan-Shadao areas, respectively. For Hsiangchiaowan-Shadao, data in 2012 were not included in the analysis due to lack of information regarding mean number of ovigerous females recorded per day.

To further access the link between intensity of human disturbance and establishment and/or spread of *A*. *gracilipes*, the generalized additive model (GAM) smoothing term with Poisson regressions was applied to investigate the correlation between average worker abundance score of *A*. *gracilipes* and proportion of human-disturbed land (note that sampling points with zero abundance score were excluded from the model). The average ant scores were considered as response variables while human disturbance levels as the fixed effect. The significance of the fixed effect was tested using the likelihood ratio test by comparing the full model with the model without the fixed effect term. Furthermore, nonparametric one-way Kruskal–Wallis test followed by pairwise Wilcoxon rank sum tests were conducted to analyse if worker abundance significantly differs across different human disturbance levels, in which human disturbance was categorized into five different levels (i.e., 0, 0–0.2, 0.2–0.4, 0.4–0.6, 0.6–0.8).

In addition, one-way Kruskal–Wallis test followed by pairwise Wilcoxon rank sum tests was also applied to analyse if worker abundance of *A*. *gracilipes* and human disturbance levels significantly differed across sampling sites. All of the statistical analyses were conducted using R program^45^ with mgcv package.

## Results

### Ant distribution and activity

**Figure 3:**
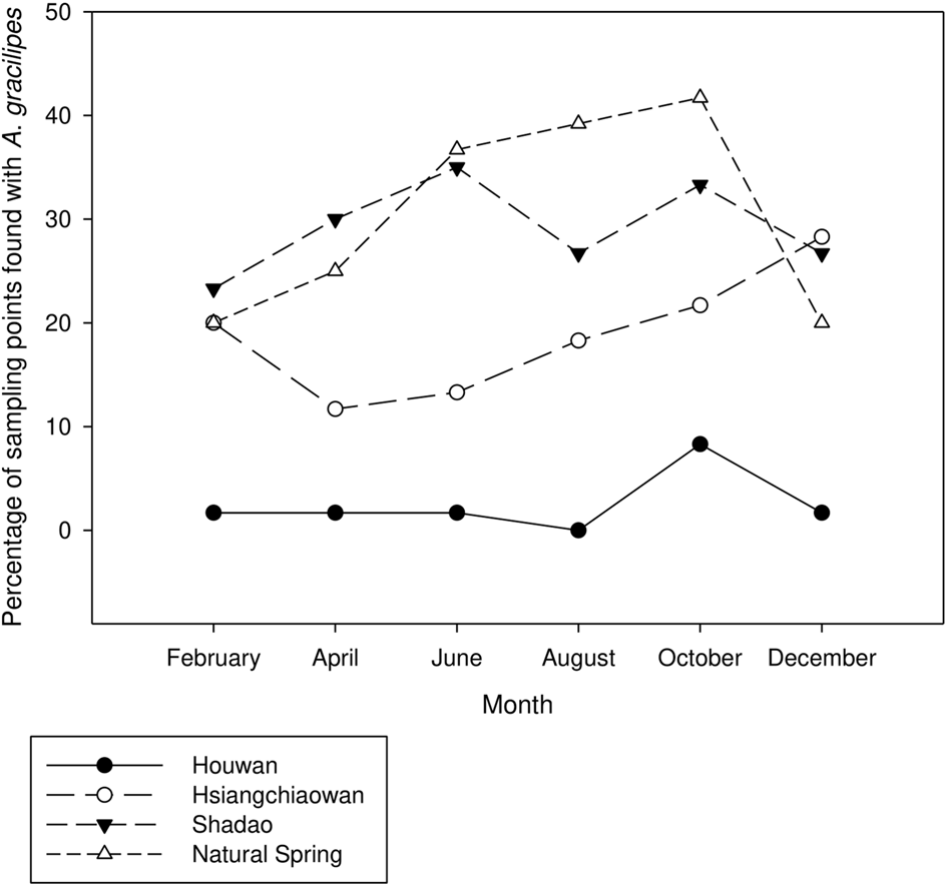
Differences in the percentage of sampling points found positive for *Anoplolepis gracilipes* at the four study sites throughout the study period.

Our distributional data showed that *A*. *gracilipes* tend to be more widespread and abundant in Shadao and Natural Spring, where more than 20% of the sampling points were found with *A*. *gracilipes* throughout the study period (Fig. 3). By contrast, the distribution of *A*. *gracilipes* was more confined in Houwan, with the majority of positive sampling points being in close proximity to a car park (see Supplementary Fig. S1.1 online).

**Figure 4:**
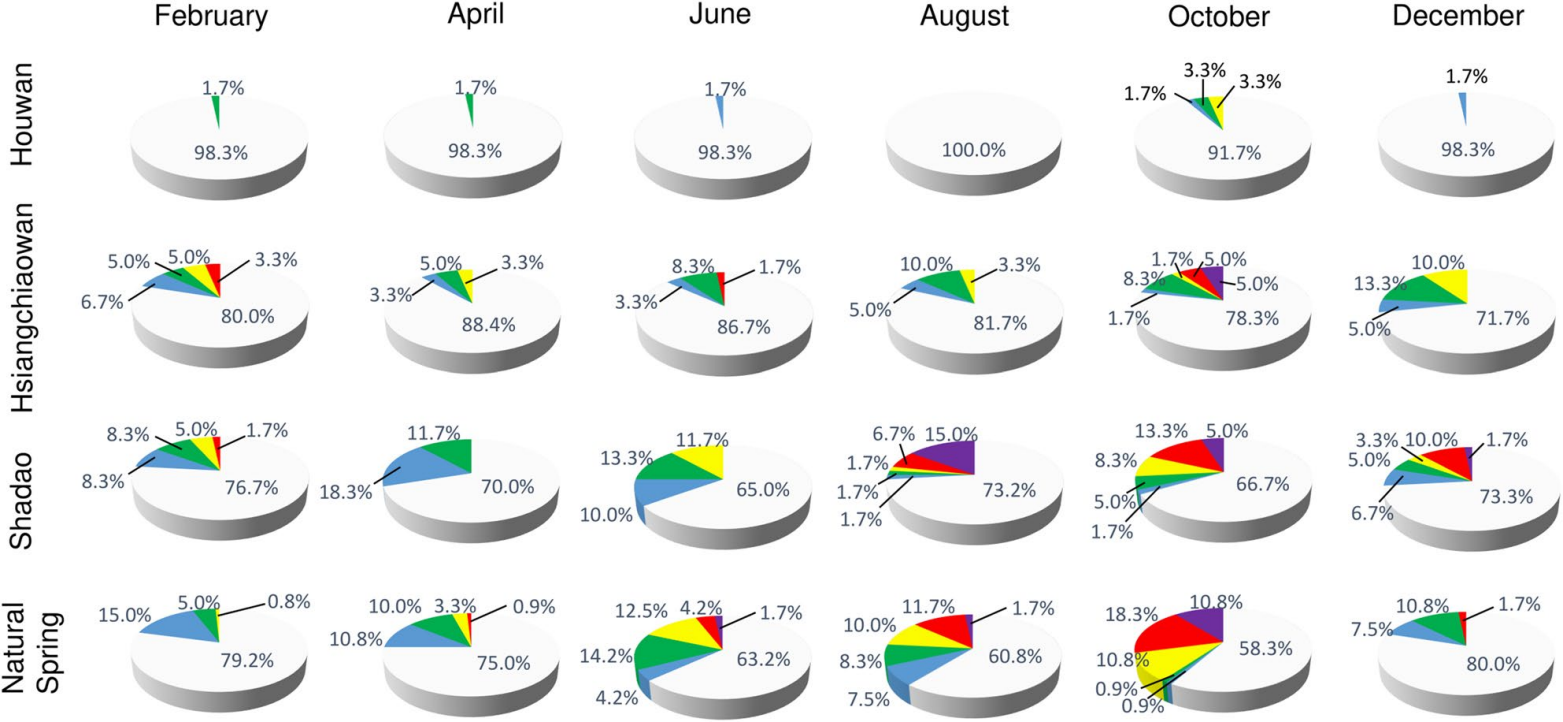
Percentage of sampling points with different worker abundance levels of *Anoplolepis gracilipes* at the four study sites throughout the study period (grey = abundance score 0; blue = abundance score 1; green = abundance score 2; yellow = abundance score 3; red = abundance score 4; purple = abundance score 5).

Throughout the study period, the distribution and activity of *A*. *gracilipes* in Houwan remained low, with positive sampling points ranging from 1.7% to 8.3%. Most of the positive sampling points were registered by an abundance score of 1 or 2 (Fig. 4). The only sampling points with an abundance score of 3 in Houwan were found during October 2017.

In Hsiangchiaowan, *A*. *gracilipes* was mostly confined to the southern part of the study site (i.e., near the fishing port), and also appeared at the northern part of the road in February 2017 (see Supplementary Fig. S1.2 online). Of the 60 sampling points, 12 (20%) were positive for *A*. *gracilipes* with abundance scores ranging from 1 to 4. In April 2017, ant distribution slightly decreased to 11.7% but *A*. *gracilipes* continuously expanded its range after April where 28.3% of sampling points were positive for *A*. *gracilipes* in December 2017 (Fig. 3). *A*. *gracilipes* was mostly confined to areas around the fishing port in Hsiangchiaowan. The ant activity was much higher in October than in other months, with 5% of the sampling points categorized with abundance scores of 4 and 5, respectively (Fig. 4).

In Shadao, *A*. *gracilipes* was mostly confined to the northern part of the road and coastal forest; however, it was also found in the middle section of the road during February 2017 (see Supplementary Fig. S1.3 online). Of the 60 sampling points, 14 (23.3%) were positive for *A*. *gracilipes,* with abundance scores ranging from 1 to 4. Overall, the distribution of *A*. *gracilipes* in Shadao fluctuated throughout the study period, and the percentage of positive sampling points ranged from 23.3 to 35.0% (Fig. 3). Ant activity was much higher in August than in other months, with 6.7% and 15.0% of sampling points being categorized with abundance scores 4 and 5, respectively (Fig. 4).

In Natural Spring, *A*. *gracilipes* was mostly confined to the northern part of the road and agricultural land during February 2017 (see Supplementary Fig. S1.4 online). Of 120 sampling points, 25 (20.8%) were positive for *A*. *gracilipes*, with abundance scores ranging from 1 to 3. *Anoplolepis gracilipes* continuously expanded its range from February to October 2017, appearing 41.7% of sampling points in October 2017 (Fig. 3). Because most ant counts were distributed in the Natural Spring I subarea, only the distribution and abundance of *A*. *gracilipes* at this subarea were provided in Supplementary Fig. S1.5 to show the pattern of ant activity in Natural Spring. Ant activity was much higher in October than in other months, with 18.3% and 10.8% of sampling points with abundance scores of 4 and 5, respectively (Fig. 4).

### Species and number of ovigerous female land crabs

Analysing the data collected by Liu^38^, we found that in Houwan all land crab species experienced a steady increase in the total number of ovigerous females except *C. carnifex,* which experienced a decrease (Table 1). By contrast, the total number of ovigerous females of *M. aubryi* decreased sharply in the Hsiangchiaowan–Shadao area over the 6-year period (2012–2017), with only three ovigerous females found in the 2017 survey (Table 2). In Houwan area, the mean number of ovigerous females recorded per day for most dominant land crab species in the 2017 survey were higher than that of 2015 survey (except *C*. *carnifex*), although no significant difference was observed (Table 1). On the other hand, in Hsiangchiaowan-Shadao area, the mean number of ovigerous females per day for *M. aubryi* in 2017 (0.8 ± 0.5) was significantly lower than that of 2015 (75.0 ± 28.8) (*t* = 2.578; d.f. = 6, *P* < 0.05) (Table 2).

**Table 1:** Total number of ovigerous females and mean number of ovigerous females recorded per day (in parentheses) for several dominant land crab species recorded during the breeding season in Houwan (2015, 2017).

Species	Observation period (number of days)		Test statistics
	August 2015 (6 days)^a^	August 2017 (6 days)	
*Cardisoma carnifex*	60 (10.0 ± 4.1)	17 (2.8 ± 1.1)	*t* = 1.704; d.f. = 10; *p* = 0.119
*Cardisoma hirtipes*	93 (15.5 ± 5.3)	147 (24.5 ± 5.3)	*t* = − 1.208; d.f. = 10, *p* = 0.255
*Sesarmops intermedium*	58 (9.7 ± 2.4)	68 (11.3 ± 2.8)	*t* = − 0.452; d.f. = 10, *p* = 0.661
*Sesarmops impressus*	94 (15.7 ± 4.2)	145 (24.2 ± 7.1)	*t* = − 1.030; d.f. = 10, *p* = 0.327

^a^Data were obtained from Liu^38^.

**Table 2:** Total number of ovigerous females and mean number of ovigerous females recorded per day (in parentheses) of *Metasesarma aubryi* at the Hsiangchiaowan–Shadao area during the peak breeding season.

Observation period (number of days)	Number of ovigerous females
September 2012 (4 days)^a,b^	> 2000
September 2015 (4 days)^a^	300 (75.0 ± 28.8)
September 2017 (4 days)	3 (0.8 ± 0.5)
Test statistic (2015 vs 2017)	*t* = 2.578; d.f. = 6; *p* < 0.05

^a^Data for 2012 and 2015 were obtained from Liu, unpublished data and Liu^38^, respectively.

^b^Data regarding mean number of ovigerous females recorded per day for the year 2012 are not available.

### Additional observation

**Figure 5:**
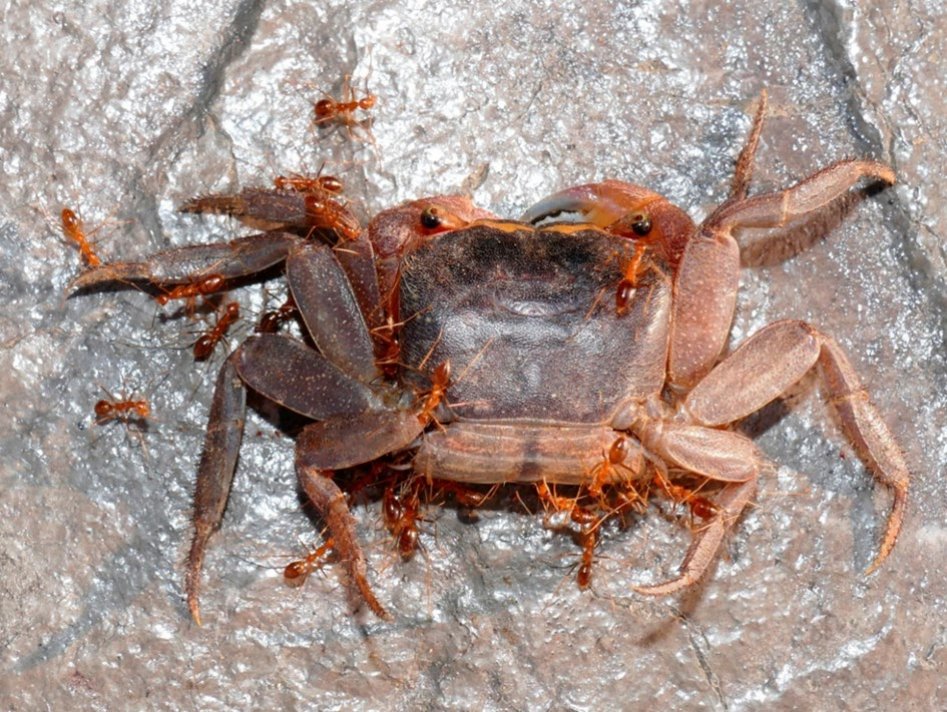
*Metasesarma aubryi* swarmed by *Anoplolepis gracilipes* workers during migration towards the ocean.

Ant attacks (Fig. 5) were only observed in the Hsiangchiaowan–Shadao area, with a total of 16 and 2 incidents in 2015 and 2017, respectively. On the contrary, ant attacks were never recorded in Houwan during the same observation periods.

### Association between abundance of *A*. *gracilipes* and human disturbance level

**Figure 6:**
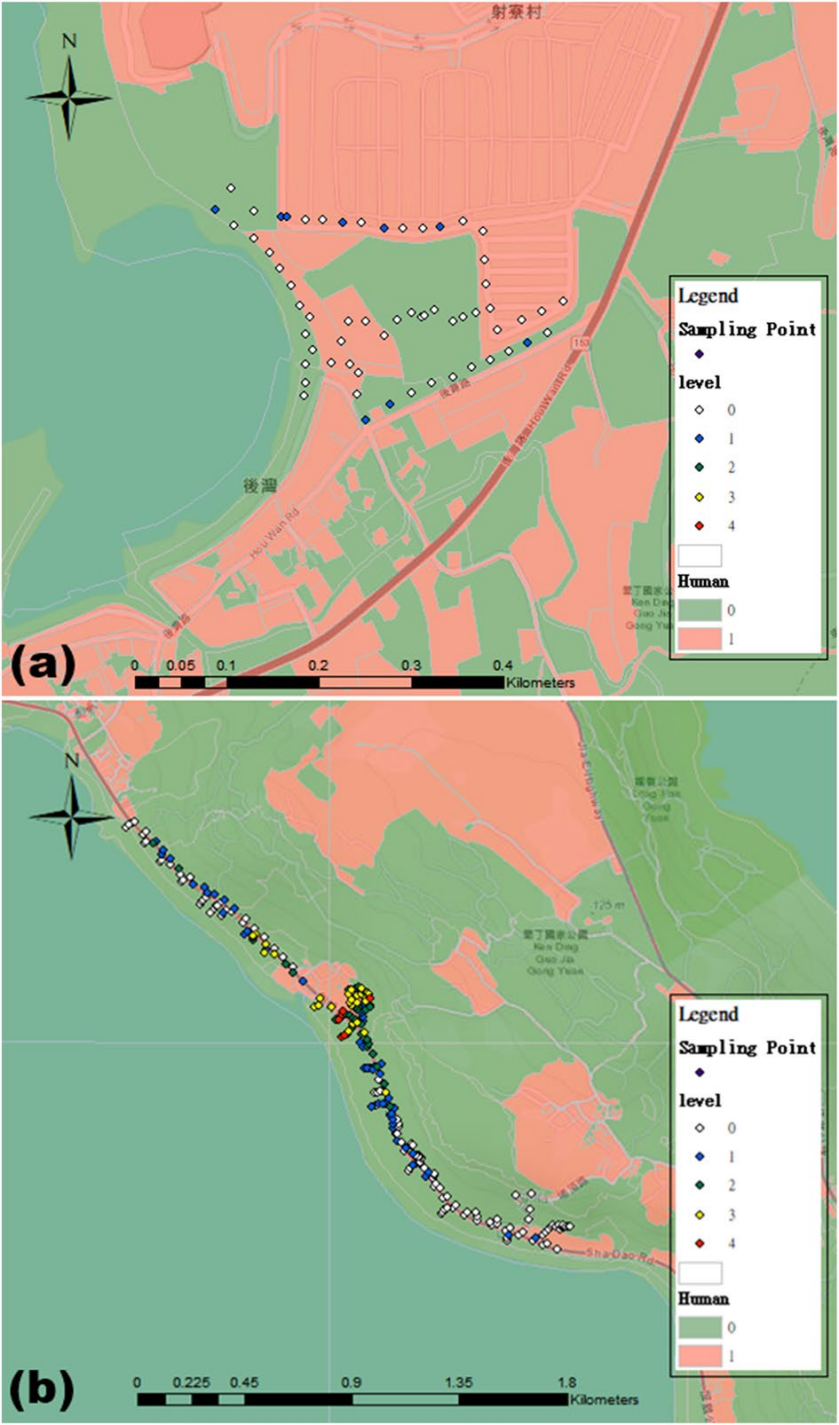
The distribution or abundance of *Anoplolepis gracilipes* in relation to proximity to human-developed land around Houwan (**a**) and Hsiangchiaowan-Shadao-Natural Spring (**b**) areas. Area of human-disturbed land and undisturbed land was represented by peach color and green color, respectively. Each sampling point was denoted by filled diamond where different colors were used to indicate quantitative differences in the average of worker abundance score (white = abundance score 0; blue = abundance score 0 < x < 1; green = abundance score 1 ≤ x < 2; yellow = abundance score 2 ≤ x < 3; red = abundance score 3 ≤ x < 4). Original copyright source: ESRI. “Topographic” [basemap]. “World Topographic Map”. February 19, 2012. http://www.arcgis.com/home/item.html?id=30e5fe3149c34df1ba922e6f5bbf808f. (October 1, 2020). The map was created using ArcGIS v.10.2, http://www.esri.com/software/arcgis/arcgis-for-desktop.

**Figure 7:**
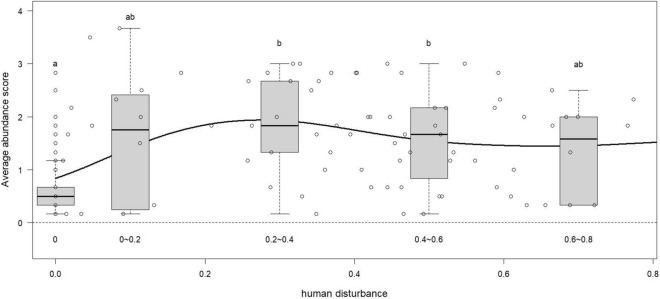
The average abundance score of *Anoplolepis gracilipes* in relation to the human disturbance level. The regression line was estimated by generalized additive models with Poisson error. Box plots labelled with different letters indicate statistical difference (*P* < 0.05) between different categories of human disturbance (Wilcoxon rank sum test).

Based on the land-use data obtained from the National Land Surveying and Mapping Center, the spatial pattern of *A*. *gracilipes* seemed to be associated with the proximity to human-developed areas (Fig. 6). Further analyses indicated that worker abundance of the ant was significantly correlated with the proportion of human-disturbed land or human disturbance (likelihood test: *X*^2^ = 21.1, *d*.*f*. = 3.821, *P* < 0.001) (Fig. 7). The worker abundance was significantly lower in the area without any human disturbance (Kruskal–Wallis test: *X*^2^ = 30.06, *d*.*f*. = 4, *P* < 0.001). Interestingly, worker abundance was highest in the area with intermediate level of human disturbance (i.e., 0.2–0.6 disturbance level) despite no significant difference compared to other levels of human disturbance (except zero disturbance).

**Figure 8:**
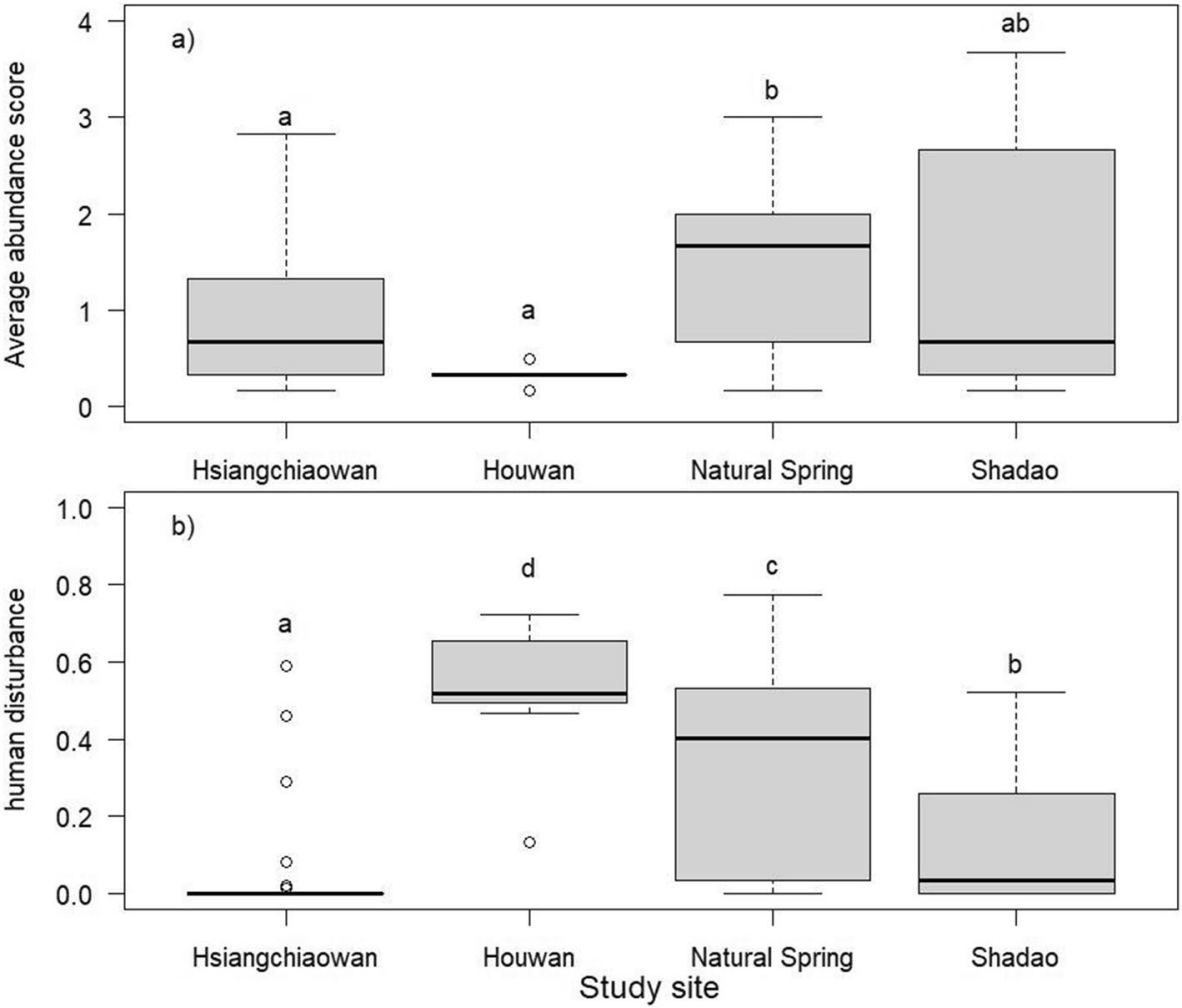
The average abundance score of *Anoplolepis gracilipes* (**a**) and human disturbance level (**b**) recorded at each study site throughout the sampling period. For each subsection of graph, box plots labelled with different letters indicate statistical difference (*P* < 0.05) between different study sites (Wilcoxon rank sum test).

In addition, differences in worker abundance were significant among the four study sites (Kruskal–Wallis test: *X*^2^ = 17.17, *d*.*f*. = 3, *P* < 0.001), with the lowest ant abundance in Houwan (Fig. 8a). Similarly, there were significant differences in the human disturbance levels across the four study sites (Kruskal–Wallis test: *X*^2^ = 43.15, *d*.*f*. = 3, *P* < 0.001), where the human disturbance was lowest in Hsiangchiaowan (Fig. 8b).

## Discussion

We conducted a field survey to characterize the status (i.e., distribution and worker abundance) of *A*. *gracilipes* and land crabs in Kenting National Park, Taiwan to demonstrate the potential negative effects of this ant on the land crab populations. The results of our survey indicated similar distribution patterns and abundances of *A*. *gracilipes* across all land crab hotpots except one. Compared with other study sites, distribution and abundance of *A*. *gracilipes* were much more confined in Houwan. The population dynamics of land crabs appear to be associated with the status of *A*. *gracilipes* within the park. In Houwan, where abundance of *A*. *gracilipes* is low, the majority of dominant land crab species underwent steady growth in the number of ovigerous females. By contrast, *A*. *gracilipes* was widely distributed with greater abundance in Hsiangchiaowan–Shadao, and the number of ovigerous *M. aubryi* declined significantly from 2015 to 2017. Our work adds to a growing body of literature that demonstrates negative impacts of *A*. *gracilipes* invasion on land crab populations. Most previous studies reporting adverse effects of *A. gracilipes* on land crab populations were from Indian and Pacific island nations^8,11,46^, while ours represents the first case in East Asia. In addition, we applied GIS-based approaches and generalized additive model to show an association of distribution of *A*. *gracilipes* and human disturbance, which constitutes one of rare datasets showing potential contribution of human land use to the occurrence and spread of the yellow crazy ant.

### Rise of *A*. *gracilipes* and decline of land crabs

Monitoring ovigerous female land crabs provides an insight into population dynamics despite lack of detailed information on the life cycle of some of the target land crab species. The population dynamics of land crabs from 2015 to 2017 in Kenting National Park seem to be tightly associated with the distribution of *A*. *gracilipes*. In Houwan, with a limited distribution and low abundance of this ant, most dominant land crab species (except for *C. carnifex*) underwent steady growth in the number of ovigerous females (Table 1). By contrast, the dominant land crab species in the Hsiangchiaowan–Shadao area, where *A*. *gracilipes* is more widespread, experienced a significant decline in the number of ovigerous females. This suggests that the presence of *A*. *gracilipes* may affect land crab populations. Although the lack of data on the population dynamic of *A*. *gracilipes* from 2012 to 2017 limits the extent to which conclusions can be drawn, additional lines of evidence are supportive to the potential of *A*. *gracilipes* negatively impacting land crab populations. For example, ant attacks had not been recorded in the Hsiangchiaowan–Shadao area until 2015^38^, which perfectly coincides with the fact that the abundance and distribution of *A*. *gracilipes* were low before 2015. It is likely that the ant’s abundance and distribution have since then reached a level that is high enough to lead to frequent encounters between *A*. *gracilipes* and land crabs. Alternatively, the lack of recorded ant attacks in Houwan may be perfectly explained by near absence or a low level of *A*. *gracilipes* abundance in this site*,* which allows the land crabs to grow over years without the ant’s interference (or at least from 2015 to 2017).

Although the present study focused on only a handful of land crab species that are dominant at the study sites, we found that such a declining trend is also true for other land crabs in Kenting National Park. Liu and Jeng^47^ reported a total of 438 ovigerous females of *Gecarcoidea lalandii* during 2003 survey in the Hsiangchiaowan–Shadao area, with 40 ovigerous females as the highest recorded number per day. Strikingly, the same survey effort in 2015 recorded only 12 ovigerous females of *G*. *lalandii*, with a maximum number of ovigerous females recorded per day of 4^38^. A similar trend was also observed in *Cardisoma rotundum*; a total of 377 ovigerous females observed during 2003, whereas only 37 ovigerous females of the same species were recorded in 2015^38^. Unlike in Hsiangchiaowan–Shadao, increases in crab populations were apparent for most dominant land crab species in Houwan prior to 2015^38,48^, and some species (e.g., *S. impressus*) even underwent a twofold increase in the number of recorded ovigerous females from 2013 to 2015. Based on the results presented in current study as well as that from previous studies, Houwan seems to serve as refugia for land crabs in the national park since land crab populations there likely face much less survival pressure exerted by the yellow crazy ant compared with other land crab hotspots.

Although a causal link between the abundance of *A*. *gracilipes* and population dynamics of land crabs could not be fully established in this study, their spatial–temporal overlap may help explain decreases in land crab populations at the hotspots with higher abundance of *A*. *gracilipes*. The yellow crazy ant is known to forage during both day and night^40^, which is also noted during our observations. The nocturnal nature of land crabs renders them highly prone to attack by *A*. *gracilipes* that forage at night, especially during their migration to the ocean for larval release (Fig. 5). Based on our observations, the aggressive behaviour of *A*. *gracilipes* towards land crabs is often triggered when the migratory pathways of ovigerous females overlap with ant territory. Similar to our findings, it was reported that a large number of red crabs were killed when ovigerous females are encountered by workers of yellow crazy ant during their annual migration on Christmas Island^49,50^. In a typical aggressive behavioural sequence, *A*. *gracilipes* workers immediately swarm any encountered land crabs and spray formic acid into their eyes and leg joints (Supplementary Movie S1). The formic acid blinds and immobilizes the land crabs, subsequently leading to death either from acid damage or dehydration. All the observed aggressive behaviours of *A*. *gracilipes* in this study were identical to those reported on Christmas Island^11,51^. Apart from the encounters with ants during migration to the ocean, we also observed *A*. *gracilipes* workers occupying land crab burrows at uplifted coral reef inside the coastal forest at Hsiangchiaowan (Liu, unpublished data), which may also represent another source of survival stress to these land crabs.

Despite observed survival threats posed by *A*. *gracilipes* invasion, at least two additional factors may have also contributed to the population decline of *M*. *aubryi* and possibly other land crab species in our study sites. First, coastal forest in the Hsiangchiaowan-Shadao area was penetrated by Provincial Highway 26 due to road and highway expansion. Since breeding season of land crabs (where ovigerous females cross the coastal road and migrate to coastline to release larvae) generally overlaps with the peak tourism season in Kenting National Park, heavy traffic volume on the road has been reported to cause significant casualty of the land crabs (i.e., roadkill)^35,52^. During a one-month survey carried out within a 3.0 km long provincial road inside Kenting National Park, a total of 663 land crabs were found dead due to roadkill^53^. Secondly, Kenting National Park is one of the most popular tourism destinations in Taiwan and attracts large numbers of visitors annually. As a result, many natural habitats of land crabs have been converted to housing, parking and commercial facilities to accommodate tourist needs and activities^35,52^. For instance, in the Hsiangchiaowan-Shadao area, land crab habitat has been dramatically altered due to construction of homestays, hotels and other related recreational facilities. In order to conserve or restore land crab populations, relevant authorities have been implementing several measures such as establishment of ecological reserve or protected area, traffic control during the peak breeding season of land crabs. Despite the effort, major declines in land crab populations at Hsiangchiaowan-Shadao have been consistently observed since 2015, suggesting that the presence of the yellow crazy ant may have negatively impacted land crab populations (especially for *M*. *aubryi*) to a notable extent.

### Anthropogenic influence and management recommendations

Although many studies focused on the effect of habitat disturbance on overall ant assemblages^54–56^, our current study is one of the few attempts to correlate worker abundance of single invasive ant species and the intensity of human disturbance. Our data show a clear trend of higher worker abundance at human-modified areas with major anthropogenic modifications such as agricultural land, farmhouses, forest edges, or fishing ports. For instance, worker abundance was highest in the Natural Spring area which mainly comprises of agricultural land and also possesses a relatively higher level of human disturbance (i.e., second highest among the four study sites). This distribution pattern of *A*. *gracilipes* indicates an apparent link between anthropogenic influence and successful establishment and subsequent spread of invasive ant species^15,57,58^. Invasions generally begin in areas with a greater level of human activity as it provides a more promising window for propagules to be introduced and possibly established in these areas^59,60^. As *A*. *gracilipes* is known to mainly reproduce by budding and hence largely relies on human factors to be introduced into distant locations^23,61^, the level of anthropogenic disturbance would perfectly explain why worker abundance was significantly lower in area without such pre-existing conditions. Apart from propagule pressure, human-related structures may also shape the abundance of invasive ants because these physical structures and space within may act as temporary shelters for ant colonies during unfavourable weather conditions^62^. Furthermore, nesting in close proximity to buildings enables ant colonies to access to diverse resources such as foods, nesting space and water from irrigation^17,63^. Our data are consistent with the notion that *A*. *gracilipes* frequently nest in or under human-made objects (e.g., wooden board, canvas, discarded cooking pot) that likely offer favourable conditions for survival^64,65^. Moreover, we have observed queens accompanied by a group of workers (some of which carried brood) travelling along water pipes from one agricultural plot to another, suggesting that human-made structures such as water pipes may also assist *A*. *gracilipes* in dispersing to distant locations (Lee, personal observation).

While the worker abundances of *A*. *gracilipes* are highly associated with the human disturbance levels in this study, the relationship does not seem to fit in a linear model. Houwan area which possesses highest level of human disturbance harbours the least number of foraging workers among the four study sites, suggesting that the intermediate level of human disturbance, instead highest, leads to highest ant abundance. Similar to our findings, Vonshak and Gordon^66^ showed that highest abundance of the invasive Argentine ant, *Linepithema humile* was observed in semi-natural sites, demonstrating that the high level of invasive ant abundance is likely driven by intermediate anthropogenic disturbance. The low abundance of *A*. *gracilipes* at the Houwan area despite higher anthropogenic disturbances may be partially explained by limited nesting site availability for *A*. *gracilipes* colonies. Previous studies suggest that *A*. *gracilipes* prefers nesting in pre-existing physical spaces such as stack of human-made objects, in cracks and crevices or within leaf litter^64,65^. Our study site in the Houwan area is mainly comprised of multiple car parks and concrete buildings where most potentially preferable nesting sites of *A*. *gracilipes* (e.g., wooden boards, piles of corrugated metal ceiling, cavities of tree trunks) are lacking. The high proportion of concrete structures at the Houwan area may not be conducive to the nesting of *A*. *gracilipes* colonies. Alternatively, the low level of *A*. *gracilipes* abundance at the Houwan area may result from a higher level of interspecific competition between *A*. *gracilipes* and other exotic ant species. This possibility was further supported by the finding that higher alien species richness is generally observed in urbanized areas, instead of semi-natural habitats^66^. Since assemblages of ant species are beyond the scope of current study, further studies (i.e., assessment of exotic ant richness among the four study sites and/or characterization of interspecific competition among these ant species at Houwan area) are needed to fill this gap.

Land crab populations in Kenting National Park may also be threatened by anthropogenic disturbances as mentioned above (e.g., habitat modification)^38^. Our findings suggest that the land crab populations in Kenting National Park may have been influenced by anthropogenic disturbance in an indirect manner. We suggest that anthropogenic disturbance may largely favour the introduction and establishment of *A*. *gracilipes* and subsequently exacerbates the effects of the ant on land crab populations via rapid population expansion of *A. gracilipes* and consequently frequent encounters between the ant and land crabs, especially during land crab’s mass migration to the ocean.

Kenting National Park houses an extraordinary level of land crab diversity. Hsiangchiaowan has the greatest land crab diversity of any coastal forest in the world, with at least 39 land crab species from 8 families formally described and recorded to date^52^. After multi-year efforts by academics and government agencies, research on the taxonomy, species distribution and breeding ecology of land crabs in the park has become internationally recognized. Land crabs are known to play multiple ecological roles at different trophic levels in an ecosystem^11,67^ and also act as a key driver in many tropical coastal ecosystems where they shape the forest composition by selective predation of seeds and seedlings, excavation of burrow, and acceleration of leaf litter breakdown^68^. Through the degradation of leaf litter, land crabs directly influence nutrient cycling process which in turn affects forest productivity^69^. With the documented importance of land crabs in various ecological processes, there is an urgent need to protect and preserve land crab populations in Kenting National Park and elsewhere.

To conclude, the key findings of current study include: (1) population dynamics of land crabs are closely associated with the status of *A*. *gracilipes* within the national park, where *A*. *gracilipes* invasion negatively impacts land crab populations; (2) worker abundance of *A*. *gracilipes* is highly correlated with the level of human disturbance, although the relationship is not linear; (3) positive interactions between anthropogenic disturbance and invasion of *A*. *gracilipes* amplify the negative impacts of this invasive ant on land crab populations. These findings highlight the necessity of control of *A*. *gracilipes* populations (in fact a chemical control program by deployment of sucrose-based liquid bait containing boric acid has been initiated in the park, Lee et al., unpublished data) but also serve as baseline information for effective evaluation of conservation interventions. Habitat restoration, coupled with removal or elimination of human-made objects that serve as potential nesting sites for *A*. *gracilipes*, seems to be among the most effective interventions as this would increase potentially favourable habitats for land crabs and meanwhile discourage the establishment for *A*. *gracilipes*.

## Supplementary Information

Supplementary Information 1.

Supplementary Video 1.

## Data Availability

Video recordings of *A*. *gracilipes* workers attacking land crab are available as Supplementary Movies S1. Spatial distribution and abundance of *A*. *gracilipes* at each sampling point throughout the survey period are provided in the Supplementary Information.

## References

[1] Holway DA, Lach L, Suarez AV, Tsutsui ND, Case TJ (2002). The cause and consequences of ant invasions. Annu. Rev. Ecol. Systemat..

[2] Moller H (1996). Lessons for invasion theory from social insects. Biol. Conserv..

[3] Hoffmann BD, Saul WC (2010). Yellow crazy ant (*Anoplolepis gracilipes*) invasions within undisturbed mainland Australian habitats: no support for biotic resistance hypothesis. Biol. Invasions.

[4] Human KG, Gordon DM (1996). Exploitation and interference competition between the invasive Argentine ant, *Linepithema humile*, and native ant species. Oecologia.

[5] Walker KL (2006). Impact of the little fire ant, *Wasmannia auropunctata*, on native forest ants in Gabon. Biotropica.

[6] Cole FR, Medeiros AC, Loope LL, Zuehlke WW (1992). Effects of the Argentine ant on arthropod fauna of Hawaiian high-elevation shrubland. Ecology.

[7] Porter SD, Savignano DA (1990). Invasion of polygyne fire ants decimates native ants and disrupts arthropod community. Ecology.

[8] Feare C (1999). Ants take over from rats on Bird Island, Seychelles. Bird Conserv. Int..

[9] Laakkonen J, Fisher RN, Case TJ (2001). Effect of land cover, habitat fragmentation, and ant colonies on the distribution and abundance of shrews in southern California. J. Anim. Ecol..

[10] Wojcik DP (2001). Red imported fire ants: impact on biodiversity. Am. Entomol..

[11] O’Dowd DJ, Green PT, Lake PS (2003). Invasional ‘meltdown’ on an oceanic island. Ecol. Lett..

[12] Ness JH, Bronstein JL (2004). The effects of invasive ants on prospective ant mutualists. Biol. Invasions.

[13] Buczkowski G, Bennett G (2008). Seasonal polydomy in a polygynous supercolony of the odorous house ant, Tapinoma sessile. Ecol. Entomol..

[14] Menke SB, Fisher RN, Jetz W, Holway DA (2007). Biotic and abiotic controls of Argentine ant invasion success at local and landscape scales. Ecology.

[15] Roura-Pascual N (2011). Relative roles of climatic suitability and anthropogenic influence in determining the pattern of spread in a global invader. Proc. Natl. Acad. Sci. USA.

[16] Bos MM, Tylianakis JM, Steffan-Dewenter I, Tsharntke T (2008). The invasive yellow crazy ant and the decline of forest ant diversity in Indonesian cacao agroforests. Biol. Invasions.

[17] Human KG, Weiss S, Weiss A, Sandler B, Gordon DM (1998). Effects of abiotic factors on the distribution and activity of the invasive Argentine ant (Hymenoptera: Formicidae). Environ. Entomol..

[18] Suarez AV, Holway DA, Case TJ (2001). Patterns of spread in biological invasions dominated by long-distance jump dispersal: insights from Argentine ants. Proc. Natl. Acad. Sci. USA.

[19] Ito F, Asfiya W, Kojima JI (2016). Discovery of independent-founding solitary queens in the yellow crazy ant *Anoplolepis gracilipes* in East Java, Indonesia (Hymenoptera: Formicidae). Entomol. Sci..

[20] Lee CC, Lin CY, Hsu HW, Yang CCS (2020). Complete genome sequences of two novel dicistroviruses detected in yellow crazy ants (*Anoplolepis gracilipes*). Arch. Virol..

[21] Wetterer JK (2005). Worldwide distribution and potential spread of the long-legged ant, *Anoplolepis gracilipes* (Hymenoptera: Formicidae). Sociobiology.

[22] Abbott KL, Greaves SNJ, Ritchie PA, Lester PJ (2007). Behaviourally and genetically distinct populations of an invasive ant provide insight into invasion history and impacts on a tropical ant community. Biol. Invasions.

[23] Haines IH, Haines JB, Cherrett JM, Williams DF (1994). The impact and control of the crazy ant, *Anoplolepis Longipes* (Jerd.), in the Seychelles. Exotic ants. Biology, impact and control of introduced species.

[24] Gerlach J (2004). Impact of the invasive ant *Anoplolepis gracilipes* on Bird Island, Seychelles. J. Insect Conserv..

[25] Hill M, Holm K, Vel T, Shah NJ, Matyot P (2003). Impact of the introduced yellow crazy ant *Anoplolepis gracilipes* on Bird Island, Seychelles. Biodivers. Conserv..

[26] Hoffmann BD, Auina S, Stanley MC (2014). Targeted research to improve invasive species management: yellow crazy ant *Anoplolepis gracilipes* in Samoa. PLoS ONE.

[27] Kaiser-Bunbury CN, Cuthbert H, Fox R, Birch D, Bunburry N (2014). Invasion of yellow crazy ant *Anoplolepis gracilipes* in a Seychelles UNESCO palm forest. NeoBiota.

[28] Wheeler WM (1909). Ants of Formosa and the Philippines. Bull. Am. Mus. Nat. Hist..

[29] Hsu, P. C. The study of habitats distribution of exotic invasive ants in Taiwan by using bait traps. Master dissertation, National Changhua University of Education (2013) (In Chinese).

[30] Tseng, H. H. The ant community structure at Kenting National Park in Taiwan. Master dissertation, National Changhua University of Education (2013) (In Chinese).

[31] Hsu JW, Shih HT (2020). Diversity of Taiwanese Brackish crabs genus *Ptychognathus* Stimpson, 1858 (Crustacea: Brachyura: Varunidae) based on DNA barcodes, with descriptions of two new species. Zool. Stud..

[32] Li JJ, Shih HT, Ng PK (2019). Three new species and two new records of *Parasesarma* De Man, 1895 (Crustacea: Brachyura: Sesarmidae) from Taiwan and the Philippines from morphological and molecular evidence. Zool. Stud..

[33] Li JJ, Hsu JW, Ng NK, Shih HT (2019). Eight new records of crabs (Decapoda, Brachyura: Sesarmidae, Varunidae) from the coasts of Taiwan. Crustaceana.

[34] Li JJ, Shih HT, Ng PK (2020). The Taiwanese and Philippine species of the terrestrial crabs *Bresedium* Serène and Soh, 1970 and *Sesarmops* Serène and Soh, 1970 (Crustacea: Decapoda: Brachyura), with descriptions of two new species. Zool. Stud..

[35] Liu, H. C. Report on the survey of ecological resources of land crab in Kenting National Park 2019–2020. Consultancy Report to Kenting National Park Headquarters, p. 155 (2020) (In Chinese).

[36] Ng PK, Li JJ, Shih HT (2020). What is Sesarmops impressus (H. Milne Edwards, 1837) (Crustacea: Brachyura: Sesarmidae)?. Zool. Stud..

[37] Shih HT, Hsu JW, Li JJ, Ng NK, Lee JH (2020). The identities of three species of *Parahelice* Sakai, Türkay & Yang, 2006 (Crustacea: Brachyura: Varunidae) from the western Pacific, based on morphological and molecular evidence. Zootaxa.

[38] Liu, H. C. Biodiversity and population abundance of the land crab fauna both at Houwan and Hsiangchiaowan-Shadao areas of the Kenting National Park. Consultancy Report to Kenting National Park Headquarters, p. 102 (2016) (In Chinese)

[39] Lin CC, Chang TW, Chen HW, Shih CH, Hsu PC (2017). Development of liquid bait with unique bait station for control of *Dolichoderus thoracicus* (Hymenoptera: Formicidae). J. Econ. Entomol..

[40] Haines IH, Haines JB (1978). Colony structure, seasonality and food requirements of the crazy ant, *Anoplolepis longipes* (Jerd.), in the Seychelles. Ecol. Entomol..

[41] Ho, P. H. Land crabs at coastal forests in Kenting National Park, p. 47 (Kenting National Park Headquarters, 2003) (In Chinese).

[42] Tung GS, Chang TP, Liu HC (2013). Monitor and comparison of terrestrial crab communities in the virgin and restoration tropical coastal forests in Kenting National Park. J. Natl. Park.

[43] National Land Survey and Mapping Center (NLSC). *The Second National Land-Use Survey* (Ministry of Interior, 2008).

[44] ESRI. *ArcGIS Desktop: Release 10* (Environmental Systems Research Institute, 2011).

[45] R Core Team. *R: A Language and Environment for Statistical Computing*; R Foundation for Statistical Computing: Vienna, Austria, 2018. Available online: https://www.R-project.org/, accessed 20 December 2018.

[46] Lester PJ, Tavite A (2004). Long-legged ants, *Anoplolepis gracilipes* (Hymenoptera: Formicidae), have invaded Tokelau, changing composition and dynamics of ant and invertebrate communities. Pac. Sci..

[47] Liu HC, Jeng MS (2007). Some reproductive aspects of *Gecarcoidea lalandii* (Brachyura: Gecarcinidae) in Taiwan. Zool. Stud..

[48] Su CY, Li JJ, Wu HJ, Chiu YW (2014). An investigation of diversity and reproduction of land crab fauna in Houwan. J. Natl. Park.

[49] Baumgartner NR, Ryan SD (2020). Interaction of red crabs with yellow crazy ants during migration on Christmas Island. Math. Biosci..

[50] Hicks JW (1985). The breeding behaviour and migrations of the terrestrial crab *Gecarcoidea natalis* (Decapoda: Brachyura). Aust. J. Zool..

[51] Green PT, O’Dowd DJ, Lake PS (1999). Alien ant invasion and ecosystem collapse on Christmas Island, Indian Ocean. Aliens.

[52] Li, J. J. & Chiu, Y. W. An atlas of land crabs on Hengchun Peninsula 2.0, p. 136 (National Museum of Marine Biology and Aquarium, 2019) (In Chinese).

[53] Liu, H. C. Land crab resource survey and management plan in Kenting National Park. Consultancy Report to Kenting National Park Headquarters, p. 88 (2010) (In Chinese).

[54] Achury R, Chacón de Ulloa P, Arcila Á, Suarez AV (2020). Habitat disturbance modifies dominance, coexistence, and competitive interactions in tropical ant communities. Ecol. Entomol..

[55] Lessard JP, Buddle CM (2005). The effects of urbanization on ant assemblages (Hymenoptera: Formicidae) associated with the Molson Nature Reserve, Quebec. Can. Entomol..

[56] Mauda EV, Joseph GS, Seymour CL, Munyai TC, Foord SH (2018). Changes in landuse alter ant diversity, assemblage composition and dominant functional groups in African savannas. Biodivers. Conserv..

[57] Bacon SJ, Aebi A, Calanca P, Bacher S (2014). Quarantine arthropod invasions in Europe: the role of climate, hosts and propagule pressure. Divers. Distrib..

[58] Tschinkel WR (1988). Distribution of the fire ants *Solenopsis invicta* and *S. geminata* (Hymenoptera: Formicidae) in Northern Florida in relation to habitat and disturbance. Ann. Entomol. Soc. Am..

[59] Pyšek P (2010). Disentangling the role of environmental and human pressure on biological invasions across Europe. Proc. Natl. Acad. Sci. USA.

[60] Rizali A (2010). Ant communities on small tropical islands: effects of island size and isolation are obscured by habitat disturbance and ‘tramp’ ant species. J. Biogeogr..

[61] Rao NS, Veeresh GK, Viraktamath CA (1991). Dispersal and spread of crazy ant *Anoplolepis longipes* (Jerdon)(Hymenoptera: Formicidae). Environ. Ecol..

[62] Gordon DM, Moses L, Falkovitz-Halpern M, Wong EH (2001). Effect of weather on infestation of buildings by the invasive Argentine ant, *Linepithema humile* (Hymenoptera: Formicidae). Am. Midl. Nat..

[63] Menke SB, Holway DA (2006). Abiotic factors control invasion by Argentine ants at the community scale. J. Anim. Ecol.

[64] Hsu PW (2020). Ant crickets (Orthoptera: Myrmecophilidae) associated with the invasive yellow crazy ant *Anoplolepis gracilipes* (Hymenoptera: Formicidae): evidence for cryptic species and potential co-introduction with hosts. Myrmecol. News.

[65] Rao NS, Veeresh GK (1991). Nesting and foraging habits of crazy ant *Anoplolepis longipes* (Jerdon) (Hymenoptera: Formicidae). Environ. Ecol..

[66] Vonshak M, Gordon DM (2015). Intermediate disturbance promotes invasive ant abundance. Biol. Conserv..

[67] Yodzis P (1984). How rare is omnivory?. Ecology.

[68] Linquist ES, Krauss KW, Green PT, O’Dowd DJ, Sherman PM, Smith TJ (2009). Land crabs as key drivers in tropical coastal forest recruitment. Biol. Rev..

[69] Koch V, Wolff M (2002). Energy budget and ecological role of mangrove epibenthos in the Caeté estuary, North Brazil. Mar. Ecol. Prog. Ser..

