# Supplementary material for: The role of anthropogenic disturbance and invasion of yellow crazy ant in a recent decline of land crab population
Source: Sci Rep. 2021 Jun 10;11:12234. doi: 10.1038/s41598-021-91732-z (PMC8192946; doi:10.1038/s41598-021-91732-z)
Supplement: Supplementary file 1 — Supplementary Information 1. [file 41598_2021_91732_MOESM1_ESM.pdf]

**Supporting Information**

Title of paper:

**The role of anthropogenic disturbance and invasion of yellow crazy ant in a recent decline of land crab population**

Ching-Chen Lee[1], Ming-Chung Chiu[1], Chun-Han Shih[1], Chin-Cheng Scotty Yang[2,3], Hung-Chang Liu[4,†], Chung-Chi Lin[1,*,†]

[1] Department of Biology, National Changhua University of Education, No. 1, Jin-De Rd., Changhua City, 50007, Taiwan

[2] Department of Entomology, Virginia Polytechnic Institute and State University, Blacksburg, VA 24061, USA

[3] Department of Entomology, National Chung Hsing University, Taichung 402204, Taiwan

[4] 53, Chenggong 11th St., Jhubei City, Hsinchu County, 302, Taiwan

† Equal contribution

* Author for correspondence:

Chung-Chi Lin, email: cclin@cc.ncue.edu.tw

**This document includes:**

Figures S1.1–S1.5

Legends for Supplementary Movie S1

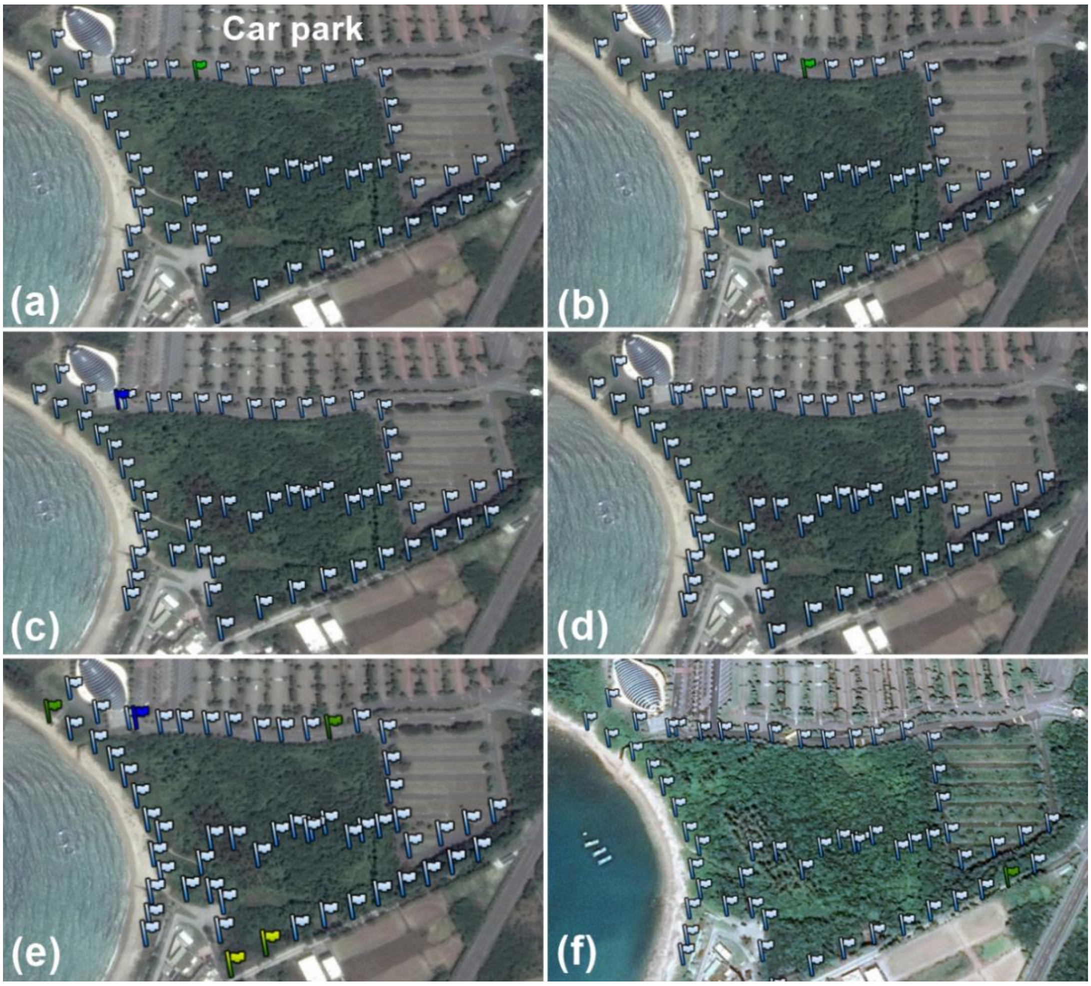


**Figure S1.1** Spatial distribution and abundance of *A*. *gracilipes* at Houwan area in February (a), April (b), June (c), August (d), October (e), and December (f) 2017. (White flag: abundance score 0; Blue flag: abundance score 1; Green flag: abundance score 2; Yellow flag; abundance score 3). The satellite imagery is from Google Earth Pro (Map data 2019 Google; https://www.google.com/maps/@22.0430792,120.6994362,331m/data=!3m1!1e3)

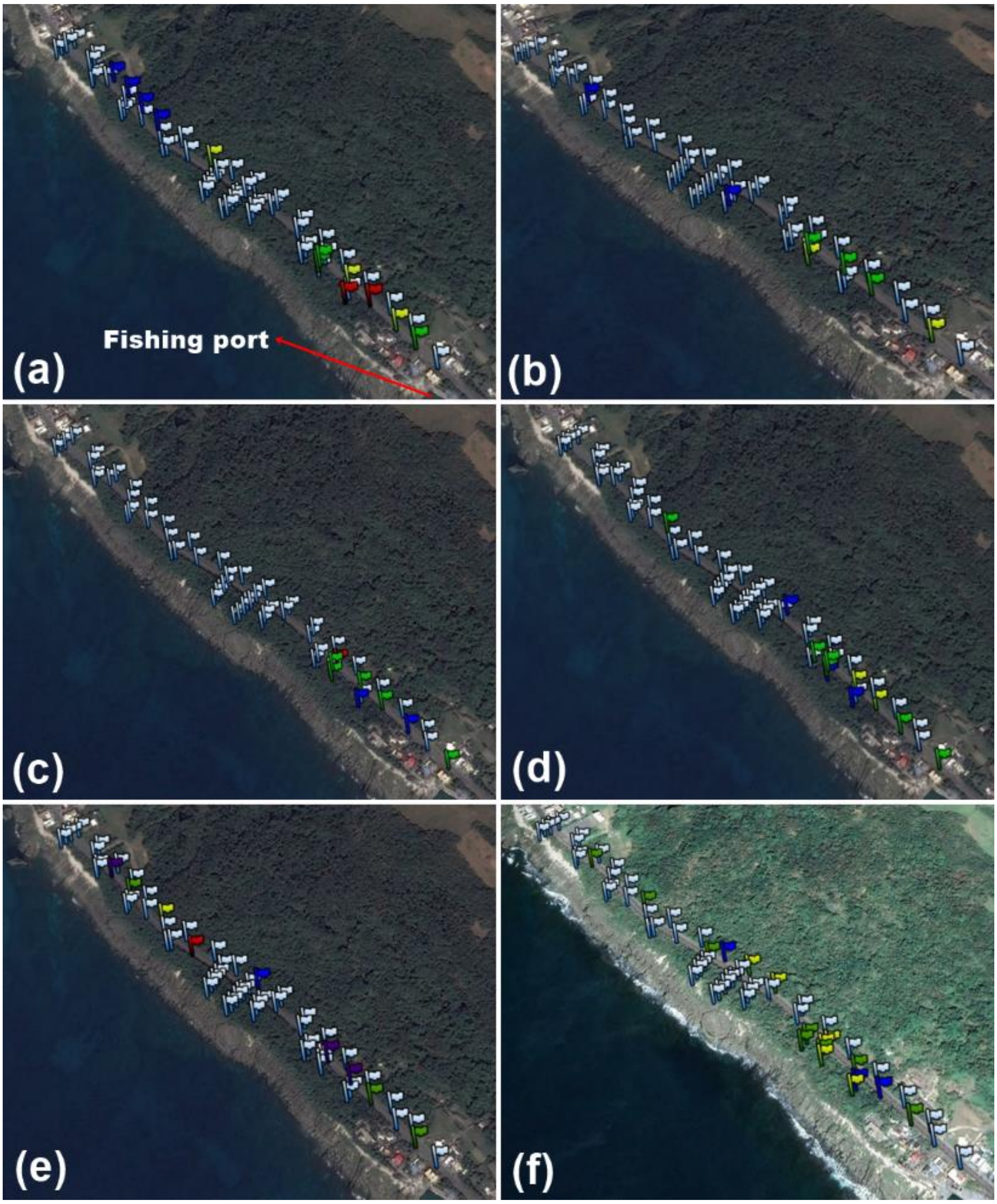


**Figure S1.2** Spatial distribution and abundance of *A. gracilipes* at Hsiangchiaowan area in February (a), April (b), June (c), August (d), October (e), and December (f) 2017. (White flag: abundance score 0; Blue flag: abundance score 1; Green flag: abundance score 2; Yellow flag; abundance score 3; Red flag: abundance score 4; Purple flag: abundance score 5). The satellite imagery is from Google Earth Pro (Map data 2019 Google; https://www.google.com/maps/@21.9289564,120.8331194,1008m/data=!3m1!)

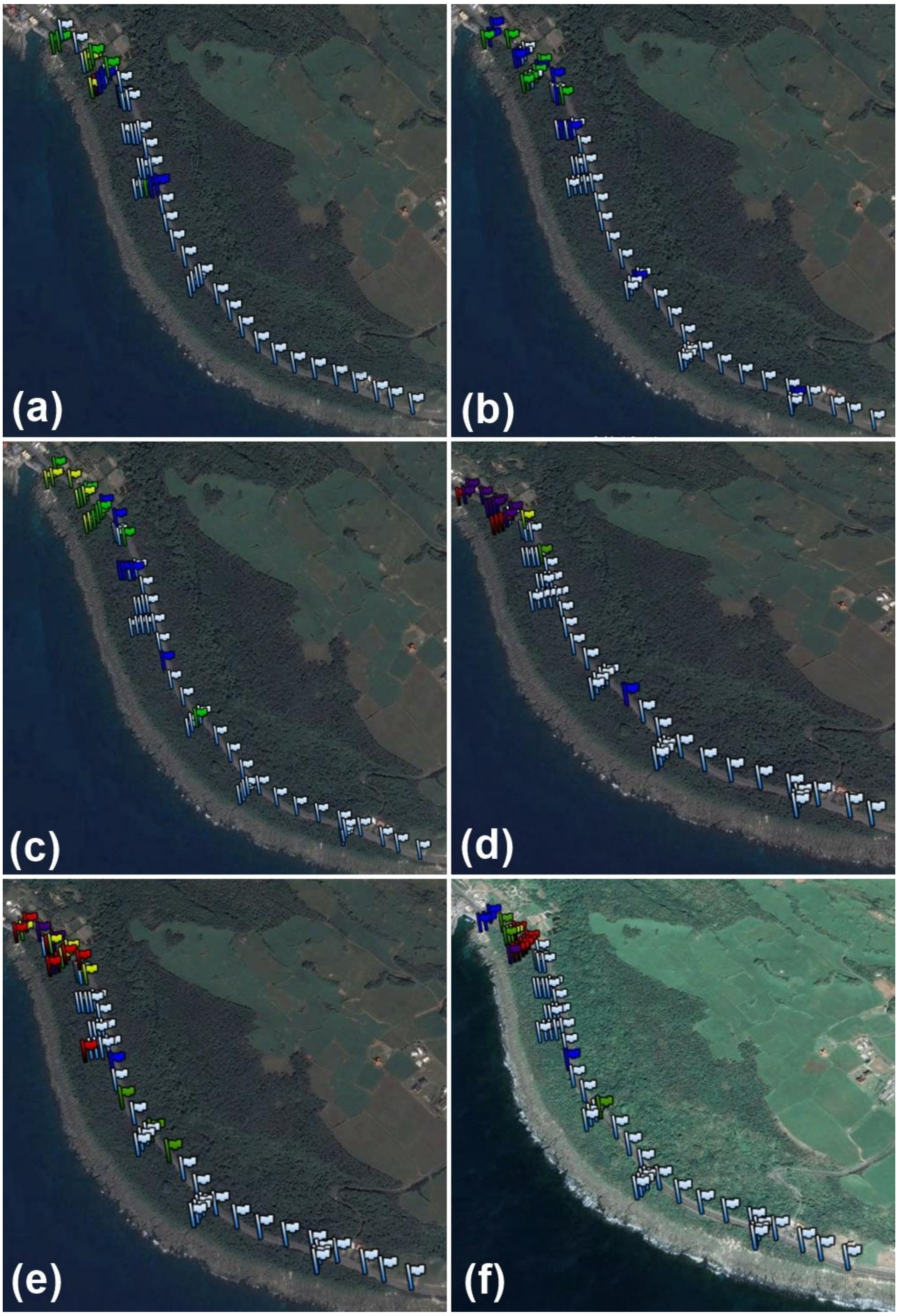


**Figure S1.3** Spatial distribution and abundance of *A. gracilipes* at Shadao area in February (a), April (b), June (c), August (d), October (e), and December (f) 2017. (White flag: abundance score 0; Blue flag: abundance score 1; Green flag: abundance score 2; Yellow flag; abundance score 3; Red flag: abundance score 4; Purple flag: abundance score 5). The satellite imagery is from Google Earth Pro (Map data 2019 Google; https://www.google.com/maps/@21.921038,120.8435391,1468m/data=!3m1!1e3)

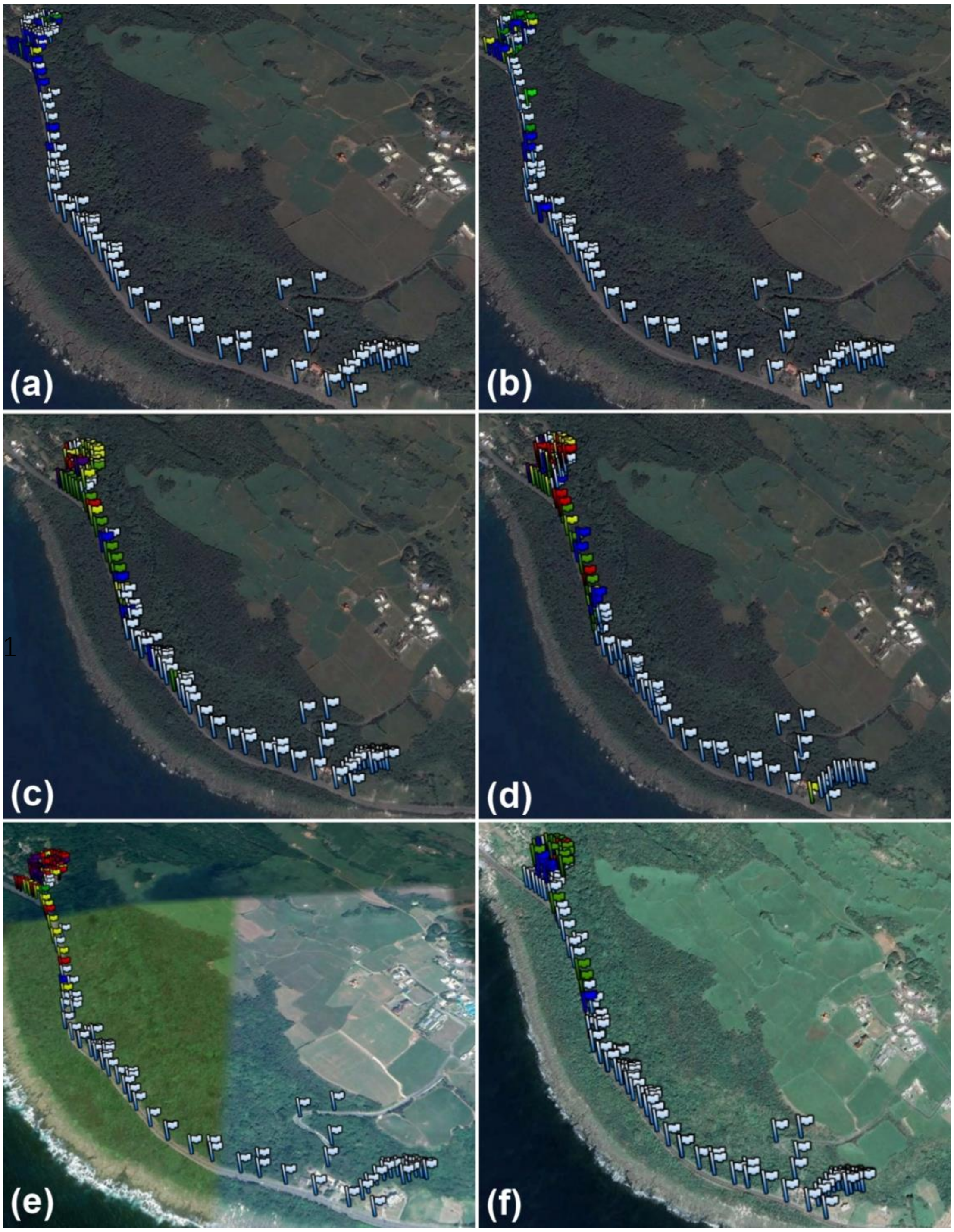


**Figure S1.4** Spatial distribution and abundance of *A. gracilipes* at Natural Spring area in February (a), April (b), June (c), August (d), October (e), and December (f) 2017. (White flag: abundance score 0; Blue flag: abundance score 1; Green flag: abundance score 2; Yellow flag; abundance score 3; Red flag: abundance score 4; Purple flag: abundance score 5). The satellite imagery is from Google Earth Pro (Map data 2019 Google; https://www.google.com/maps/@21.9212603,120.844852,1468m/data=!3m1!1e3)

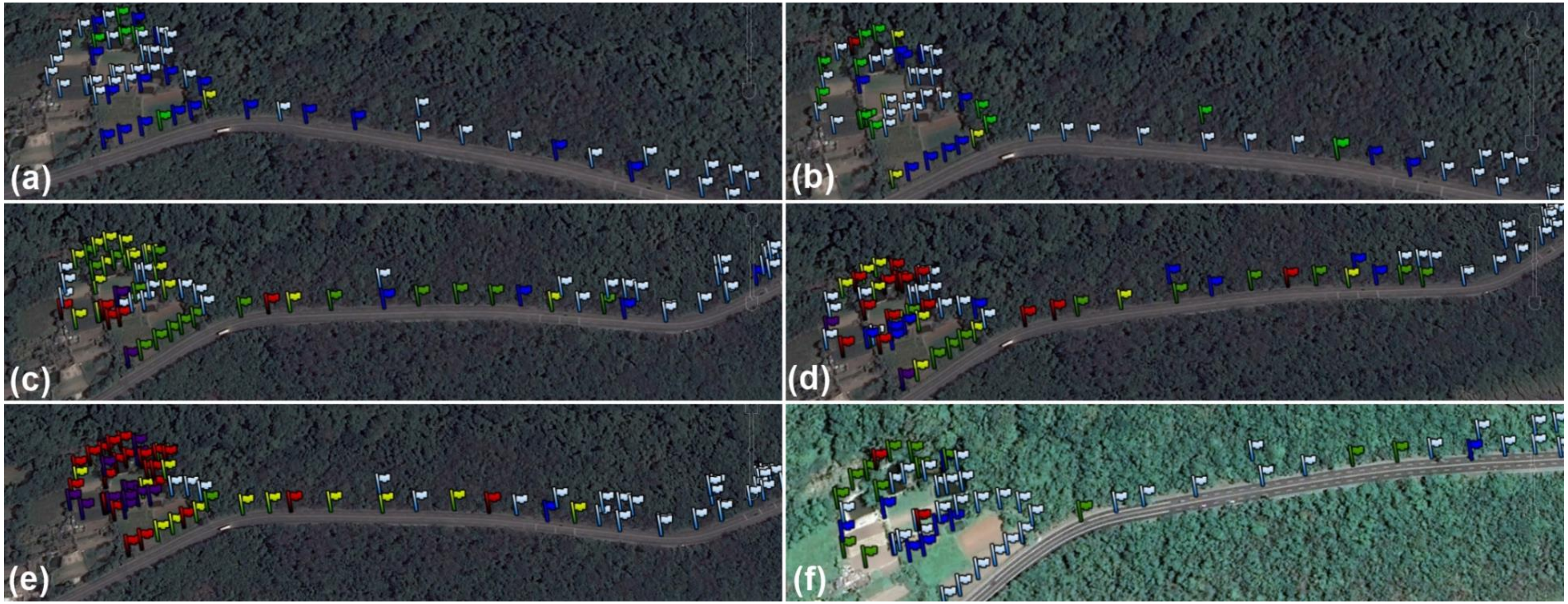


**Figure S1.5** Spatial distribution and abundance of *A. gracilipes* at Natural Spring area (only first 60 sample points included) in February (a), April (b), June (c), August (d), October (e), and December (f) 2017. (White flag: abundance score 0; Blue flag: abundance score 1; Green flag: abundance score 2; Yellow flag; abundance score 3; Red flag: abundance score 4; Purple flag: abundance score 5). The satellite imagery is from Google Earth Pro (Map data 2019 Google; https://www.google.com/maps/@21.9220601,120.8350707,777a,35y,90h/data=!3m1!1e3)

**Legend for Supplementary Movie**

**Movie S1.** Worker ants of *A. gracilipes* swarming over and biting the leg joints of a land crab (*Geosesarma hednon*).
